# Non-hormonal pharmacological treatments for benign prostatic obstruction: a systematic review of trials published between 2005 and 2025

**DOI:** 10.3389/fsurg.2026.1898220

**Published:** 2026-10-28

**Authors:** Jessica Diong, Andrea Ong, Sohani N. Dassanayake, Alexis Wong, Nadine McCauley, Lazaros Tzelves, Patrick Juliebø-Jones, Bhaskar K. Somani

**Affiliations:** 1 Department of Urology, Victoria Hospital Kirkcaldy, Scotland, United Kingdom; 2 Department of Urology, Royal Preston Hospital, Preston, United Kingdom; 3 Department of Urology, University Hospital Southampton, Southampton, United Kingdom; 4 Department of Urology, Manchester Royal Infirmary, Manchester, United Kingdom; 5 Department of Urology, Southmead Hospital, Bristol, United Kingdom; 6 Second Department of Urology, Sismanoglio Hospital, Athens, Greece; 7 Department of Urology, Haukeland University Hospital, Bergen, Norway; 8 Department of Urology, Haraldsplass Deaconess Hospital, Bergen, Norway

**Keywords:** benign prostatic hyperplasia, LUTS (lower urinary tract symptoms), non-hormonal, pharmacology, prostate

## Abstract

**Background and objective:**

Benign prostatic obstruction (BPO) is a common condition primarily affecting men over the age of 60. The associated lower urinary tract symptoms (LUTS) vary from mild to severe and are often pervasive, significantly impacting quality of life. There have been many pharmacological treatments developed to treat BPO. This review aims to assess the efficacy of different non-hormonal, pharmacological treatment options at alleviating the symptoms of BPO.

**Methods:**

A descriptive systematic review of studies published between January 2005 and March 2025 was conducted across MEDLINE, Cochrane, Web of Science and Scopus according to PRISMA guidance. Studies evaluating non-hormonal pharmacological therapies in adult men with BPO were included. Primary outcomes were IPSS, IPSS QoL, Qmax, and PVR. Secondary outcomes were adverse effects and rates of discontinuation. Treatment arms were grouped by pharmacological class, monotherapy and combination therapy, and arm-level outcomes were summarised descriptively without meta-analysis.

**Results:**

42 studies involving 10,066 participants were included. 7,916 participants were allocated to receive the following treatments; a1-adrenergic receptor antagonists (a1-blockers) (*n* = 1,911), phosphodiesterase type 5 inhibitors (PDE5-i) (*n* = 4,579), combination of a1-blockers and PDE5-i (*n* = 1,206), and an antimuscarinic in combination therapy (*n* = 220). The use of a1-blockers, PDE5-i, the combination of a1-blockers and PDE5-i, and its use alongside antimuscarinics yielded reductions in IPSS. Improvements in Qmax were modest with monotherapy. Use of a1-blocker + PDE5-i combination therapy showing the greatest improvements across all primary outcome parameters.

**Conclusion:**

In contemporary trials, non-hormonal pharmacological therapies were associated with symptomatic improvement in men with LUTS secondary to BPO, with the largest numerical improvements in arms combining an a1-blocker with a PDE5-i. These unadjusted, descriptive observations do not establish comparative superiority. The evidence base remains limited by short follow-up and the near absence of progression endpoints; future trials should report long-term outcomes such as acute urinary retention and the need for surgery, and should stratify by patient characteristics such as storage symptoms, erectile dysfunction and prostate volume.

## Introduction

Histological evidence has identified benign prostatic hyperplasia (BPH) in 50%–60% of men aged 60–70 years, increasing to 80%–90% of men over the age of 70 (1). According to the World Health Organisation, the global population aged 60 and above will double to 22% by 2050 (2). BPH can result in obstruction to urine flow, also known as benign prostatic obstruction (BPO). Lower urinary tract symptoms (LUTS) secondary to BPO can significantly impact quality of life and place a considerable burden on healthcare systems as there is an increased risk of urinary retention and infections (1). Although surgical intervention remains an effective treatment option, many patients are either unsuitable for surgery or prefer less invasive management. Drugs such as 5-alpha reductase inhibitors work by reducing prostate volume via hormonal mechanisms. However, the side effects from them are often poorly tolerated by patients (3). The medical management of LUTS secondary to BPO is a mature field, and the efficacy of a1-blockers is well established and summarised in international guidelines (3, 4) and earlier meta-analyses (5). However, the range of non-hormonal options has changed over the last two decades, with the approval of tadalafil 5 mg once daily for LUTS, the wider use of more uroselective a1-blockers such as silodosin, and growing use of combination regimens. Many previous systematic reviews have focused on a single drug class or a single pairwise comparison (6), and fewer have summarised, across classes, the tolerability and discontinuation data from contemporary trials, or the extent to which these trials report clinically meaningful long-term outcomes such as acute urinary retention or the need for surgery. This systematic review therefore aims to summarise the efficacy and tolerability of non-hormonal pharmacological treatments for LUTS secondary to BPO reported in trials published since 2005, and to identify the evidence gaps that future studies should address. The primary outcomes were change in LUTS measured through maximum urinary flow rate (Qmax), post-void residual volume (PVR), International Prostate Symptom Score (IPSS) and IPSS Quality of Life Sub-score (IPSS QoL). Secondary outcomes included adverse effects and treatment discontinuation rates.

## Methodology

A systematic review was conducted following Preferred Reporting Items for Systematic Reviews and Meta-Analyses (PRISMA) guidelines (7). A comprehensive literature search was conducted via MEDLINE (via PubMed), Cochrane CENTRAL, Web of Science, and Scopus, for studies published between January 2005 and March 2025. This period was chosen to capture contemporary trials, including those evaluating PDE5-i and combination therapy. As a consequence, the pivotal placebo-controlled a1-blocker registration trials, most of which were published before 2005, were outside the scope of this review. Data saturation was deemed to be satisfactory as searches from additional databases did not identify further relevant studies. The key search terms included “non-hormonal medical treatment”, “non-hormonal medical therapies”, “phosphodiesterase-5-inhibitors”, “anti muscarinic”, “alpha-adrenoceptor blockers”, “benign prostatic hyperplasia”, and “benign prostatic obstruction”. Boolean operators “AND” and “OR” were used to combine terms. Only studies published in English were included. The full search strategy for each database, including all search terms, limits and the date of the final search, is provided in Supplementary Table 3.

Inclusion criteria:

Randomized controlled trials (RCTs), quasi-experimental studies, and controlled clinical trials evaluating non-hormonal pharmacological treatments for BPO in adult men, regardless of symptom severity.

Exclusion criteria:

Post hoc analysis, case reports, reviews, editorials, conference abstracts without full data, and studies not reporting clinical outcomes relevant to BPO were excluded. Studies including hormonal therapies or patients with a diagnosis of prostate cancer or other significant urological pathologies were excluded. *In vitro* studies and papers that are not available in the English language were excluded.

Screening of identified studies was conducted by two independent reviewers (A.O. and A.W.). Duplicates were excluded and titles and abstracts were screened against the eligibility criteria. Full articles were reviewed by three of the authors (A.O., J.D., and A.W.) and articles that did not meet inclusion criteria were excluded (Figure 1). Disagreements were resolved by discussion, with a senior author consulted where consensus could not be reached.

**Figure 1:**
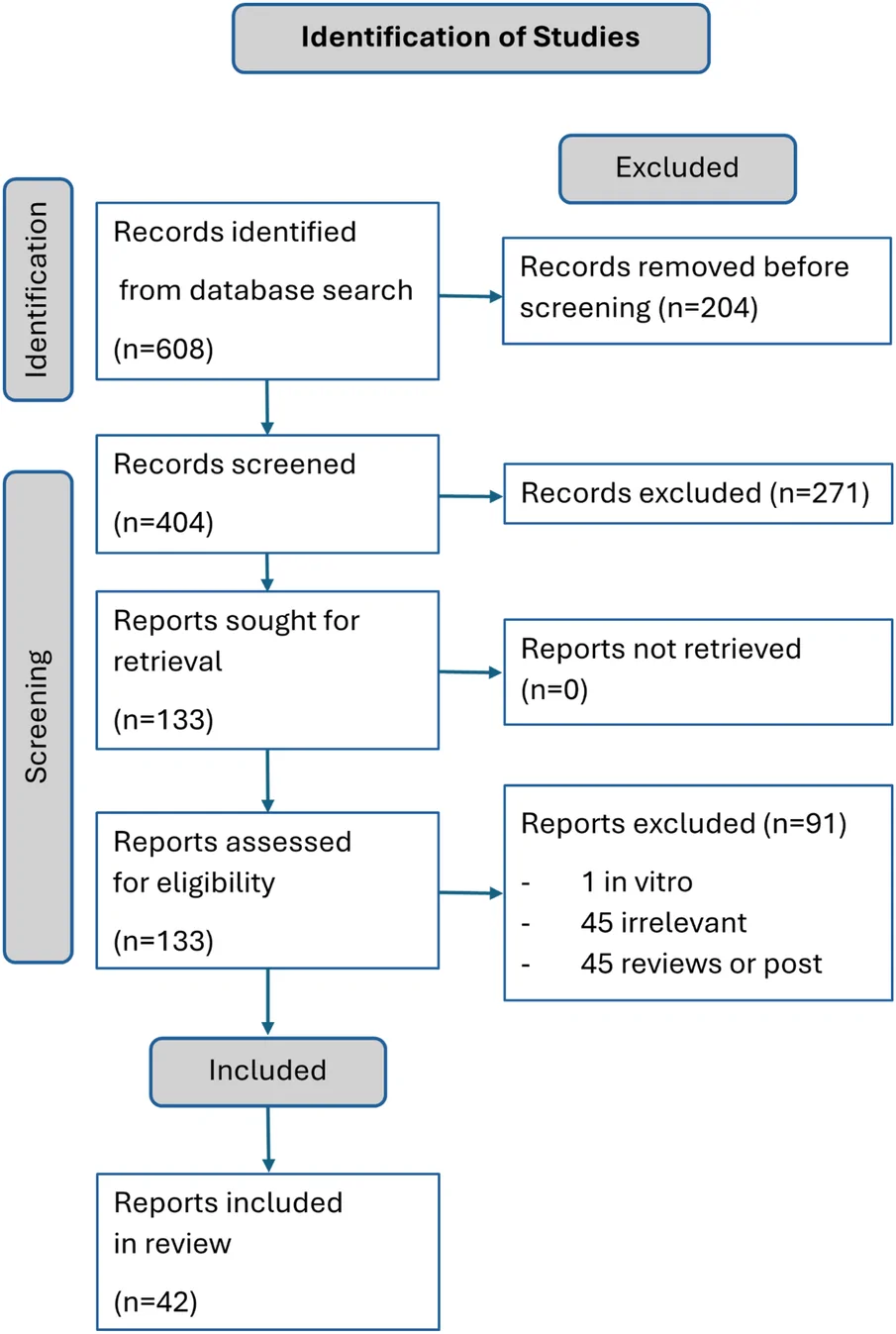
PRISMA flow diagram showing the search from MEDLINE (via PubMed), Cochrane CENTRAL, Web of Science, Scopus, and listed references from January 2005 to the date of final search March 2025.

Data extracted included study design, year of publication, country, sample size, intervention, duration of study, participant characteristics, drug class, dosage, outcomes and adverse events/side effects. Treatment arms were subsequently grouped by drug class and categorised as monotherapy or combination therapy. Results were analysed using Microsoft Excel, and Box-and-Whisker plots were produced for each outcome. Owing to substantial heterogeneity between studies in treatment regimens, participant characteristics, baseline symptom severity, and follow-up duration, outcomes were summarised descriptively. For each treatment arm, the mean change from baseline to the end of treatment was extracted for each outcome, together with the baseline value; where only baseline and end-of-treatment values were reported, the change was calculated as the difference between them. For each treatment group, results were summarised as the unweighted average of the arm-level mean changes and their range. Because treatment groups combined different agents, doses and formulations, these summaries are descriptive and are not pooled estimates of efficacy; no meta-analysis was performed. No formal between-group statistical comparisons were performed.

A risk of bias assessment was conducted independently by 2 reviewers (A. O. and J. D.) using the RoB-2 tool and ROBINS-I, according to study design (8, 9).

This review was registered retrospectively on the Open Science Framework (OSF; DOI 10.17605/OSF.IO/SV2Z7). The full protocol, including the eligibility criteria, search strategies, data extraction items, risk of bias assessment and planned descriptive synthesis, has been deposited in the same OSF project. We acknowledge that retrospective registration is a limitation of this review.

## Results

A total of 608 results were identified from the initial literature search. 42 papers were eligible for inclusion (10–51). Included articles reported on 10,066 participants, with a total of 7,916 participants allocated to receive the following treatments; a1-adrenergic receptor antagonists (a1-blockers) (*n* = 1,911), phosphodiesterase type 5 inhibitors (PDE5-i) (*n* = 4,579), combination of a1-blockers and PDE5-i (*n* = 1,206), and an antimuscarinic in combination therapy (*n* = 220). A singular treatment arm (*n* = 37) was randomised to solifenacin monotherapy with 25 participants completing the trial (18). Due to the small sample size and lack of poolable data within the identified literature, this treatment arm was excluded from this descriptive analysis.

Studies included originated from multiple countries, including Argentina, Japan, South Korea, Taiwan, Brazil, China, Egypt, Germany, India, Australia, Austria, Belgium, France, Greece, Italy, Mexico, Netherlands, Poland, Iran, United States of America, Canada, and Turkey with 53.4% of them conducted as multicentre studies. The mean age of participants was 62.6 years. Supplementary Table 1 summarises the characteristics of the included studies, including the drug, dose and formulation used in each treatment arm and the baseline IPSS, Qmax and PVR values. Throughout the Results, “mean change” refers to the unweighted average of the arm-level mean changes within each treatment group, and ranges refer to the lowest and highest arm-level values.

### Alpha-1 adrenergic antagonist (a1-blocker)

A total of 1,911 participants were allocated to receive a1-blocker and 1,796 of these participants completed their respective trials (94.0%). The majority of participants received tamsulosin (56%). PVR mean change was measured in 25 of the 30 treatment arms. Two treatment arms found a rise in PVR. One where participants were switched from tamsulosin to silodosin found PVR increased by 4.4 mL, and another group allocated to silodosin found a rise of 2.6 mL. All other treatment arms showed a decrease in PVR. The mean change in PVR was—24.8 mL, with a range of −63.3 mL to +4.4 mL.

The maximum urine flow rate (Qmax) mean change was measured in 25 of the 30 treatment arms. An improvement in Qmax was seen in 100% of the arms. The mean Qmax change was an increase of 2.49 mL/s (range 0.1 mL/s to 8.1 mL/s). All treatment arms recorded an improvement in IPSS. The mean improvement in IPSS was a reduction of 7.08 points (range: 2.7–16.2 points). IPSS QoL mean change was reported in 17 of the 30 treatment arms, each showing an improvement in IPSS QoL. The mean change was a reduction of 1.48 points (range: 0.2–4.1 points). Only 19 (1.2%) participants discontinued medications due to adverse effects. Overall, a1-blockers produced consistent improvements across Qmax, PVR, IPSS, and IPSS QoL, with low discontinuation rates due to adverse events (*n* = 30).

### Phosphodiesterase type-5 inhibitor (PDE5-i)

There were 4,579 participants given a PDE5-i monotherapy and 87.7% (*n* = 4,017) completed their respective trials. PVR mean change was measured in 27 out of 39 treatment arms. In 85% (23 arms), it showed an improvement in PVR with a mean reduction of 10.2 mL (range: −48.9 mL to +4.7 mL). The change in Qmax was measured in 31 out of 39 treatment arms. In 93.5% (29 out of the 31 arms), it showed an increase in Qmax, with a mean change of +1.73 mL/s (range: −1 mL/s to +7.2 mL/s).

Change in IPSS was measured in 39 out of 39 treatment arms. In 97.4% (38 arms) PDE5-i showed a reduction in total IPSS with a mean reduction of 5.03 points (range +0.3 to −9.2). Of note, one study investigating Sildenafil (25–100 mg PRN per week) showed a slight worsening in mean IPSS (38). IPSS QoL mean change was recorded in 29 out of 39 treatment arms. All treatment arms reported an improvement in IPSS QoL, showing a mean reduction of 1.12 points (range: −4 to −0.1). The discontinuation rate was 3.2% (*n* = 146) due to adverse effects. Similar to a1-blockers, PDE5-i showed improvements across Qmax, PVR, IPSS, and IPSS QoL and was well tolerated by participants.

### Combination: a1-blocker + PDE5-i

A total of 1,206 participants were given a combination of an a1-blocker and PDE5-i. This combination therapy had a completion rate of 87.9% (*n* = 1,060). All 12 treatment arms that measured PVR showed an improvement. The mean change was a reduction of 29.3 mL (range: −8.7 mL to −79.5 mL). All of the 15 arms that measured the change in Qmax showed an improvement, with a mean change in rise of Qmax of 4.01 mL/s (range 1 mL/s to 8.7 mL/s). There was an improvement in IPSS in all treatment arms with a mean reduction of 7.68 points (range 2.8 to 12.3 points). IPSS QoL mean change measured in 9 out of 17 treatment arms. An improvement was shown in all arms with a mean reduction of 2.26 points (range: 1.3–4.5 points). Only 27 (2.2%) participants discontinued treatment due to adverse effects. Overall, combination therapy with a1-blockers and PDE5-i showed significant improvement in Qmax, PVR, IPSS, and IPSS QoL. The medication was well tolerated by participants.

### Combination: antimuscarinic + a1-blocker

A total of 220 participants were included in the two treatment arms. There was a reduction in PVR when tolterodine was combined with both tamsulosin (−17.3 mL/s) and with doxazosin (−19.1 mL/s). Qmax increased with both tamsulosin + tolterodine and doxazosin + tolterodine by 3.4 mL/s and 3.8 mL/s respectively. There was a greater reduction in IPSS and IPSS QoL in the doxazosin + tolterodine (IPSS: −8.9, IPSS QoL: −1.8), compared to tamsulosin + tolterodine (IPSS: −5.4, IPSS QoL: −1.1). Discontinuation rate was 12.7% (28 participants) due to adverse events, all remaining participants (*n* = 192) completed their trial.

This single study suggests that Qmax, IPSS and IPSS QoL improves when tolterodine is combined with an a1-blocker. However, this treatment was less well tolerated than a1-blocker taken without antimuscarinic.

### Safety

The reported adverse effects were highly heterogeneous across the included studies. Therefore, the most frequently reported adverse events within each treatment group were summarised to facilitate comparison (see Figure 2). Discontinuation due to adverse events occurred in 19 participants (1.2%) receiving a1-blocker monotherapy, 146 (3.2%) receiving PDE5-i monotherapy, 27 (2.2%) receiving a1-blocker plus PDE5-i, and 28 (12.7%) receiving antimuscarinic combination therapy. Headache was the most frequently reported adverse event overall, nasopharyngitis was common with both PDE5-i and a1-blockers, and dry mouth was the most frequent adverse event with antimuscarinic combination therapy. Serious adverse events were rare. Two deaths were reported, both in tadalafil arms: one was considered unrelated to treatment and one, due to acute myocardial infarction, was considered possibly related (31, 40). One acute myocardial infarction was reported in a tamsulosin arm (42). No cases of acute urinary retention were reported with antimuscarinic combination therapy.

**Figure 2:**
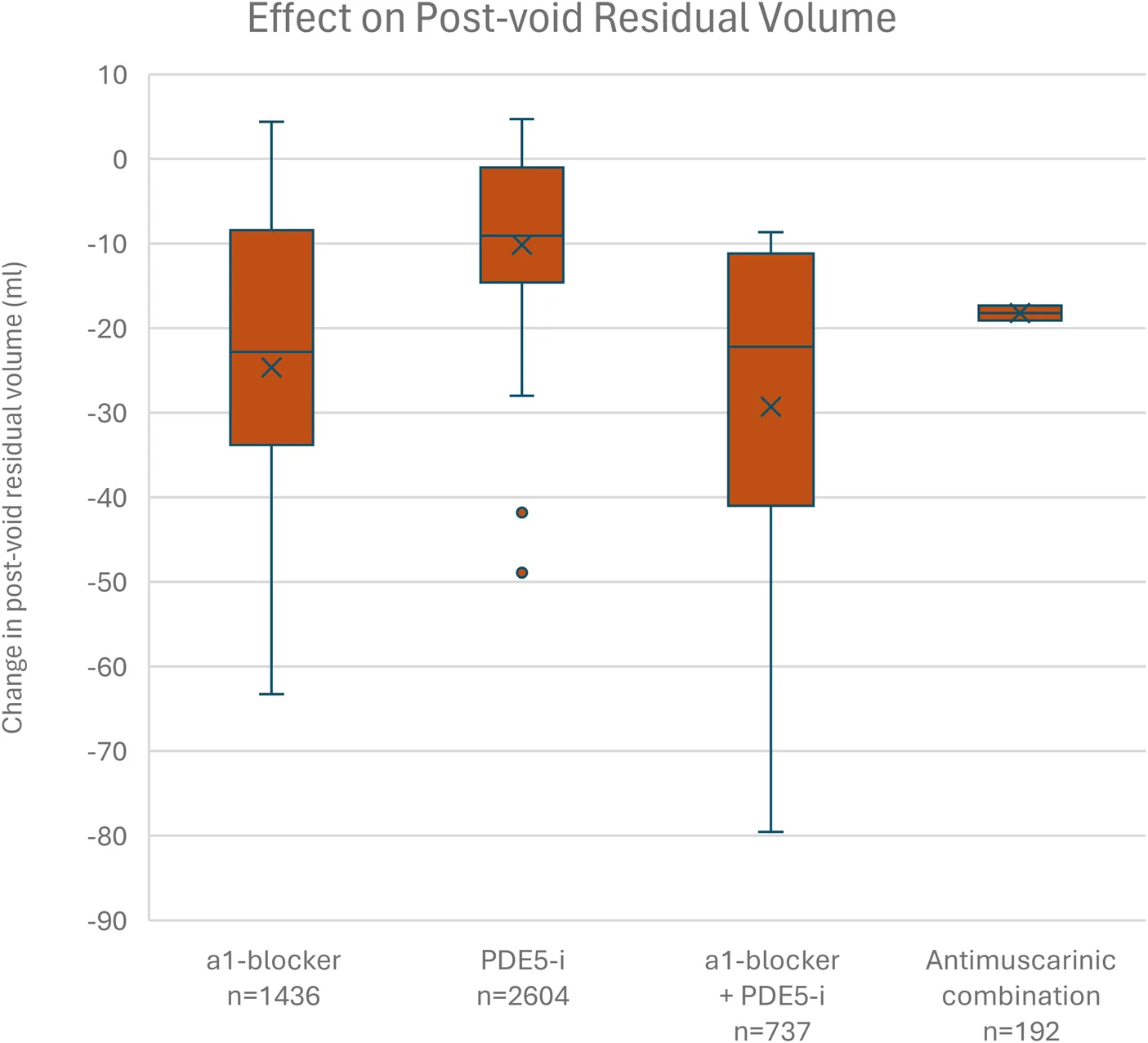
Graphs showing the recorded adverse effects from each of the treatment groups.

### Risk of bias analysis

The risk of bias of each paper was assessed by authors AO and JD. The RoB-2 tool was used for 36 papers and the ROBINS-I tool was used for the remaining 6 papers. These can be visualised in Supplementary Table 2. Overall, 6 papers had high risk of bias, and 23 papers were found to be at moderate risk of bias. The remaining 13 papers had low risk of bias.

## Discussion

In this systematic review of 42 studies including 10,066 participants, pharmacological therapy for LUTS secondary to BPO was consistently associated with improvements in both patient-reported symptom burden (IPSS) and objective urinary flow parameters (10–51).

### Primary outcomes

The primary outcomes of interest were measured using validated patient reported questionnaires (IPSS and IPSS QoL) and urinary flow parameters (Qmax and PVR), allowing for both subjective and objective monitoring of drug effect. While the measurement of treatment effect on prostatic hyperplasia is prostate volume, few clinical trials use this parameter as it requires an invasive procedure or exposure to radiation. Additionally, this would not account for improvement in LUTS by mechanisms such as smooth muscle relaxation of the prostate and bladder neck. This systematic review found that treatment arms combining an a1-blocker and a PDE5-i demonstrated the largest mean improvements across the reviewed studies in IPSS, IPSS QoL, PVR, and Qmax. In contrast, PDE5-i monotherapy generally demonstrated the smallest mean improvements in these outcomes. a1-blocker monotherapy showed consistent improvements across both subjective symptom scores and objective urinary flow parameters and remains aligned with current European Association of Urology (EAU) and American Urological Association (AUA) recommendations as first-line therapy for men with LUTS secondary to BPO (3, 4). Given the substantial heterogeneity between studies, these observations should be interpreted as descriptive trends rather than direct comparative evidence of superiority. Evidence regarding antimuscarinic combination therapy was limited to a small number of participants and studies, restricting meaningful comparisons with other treatment approaches.

### Adverse effects

The secondary aim of this review was to assess the tolerability of the treatment regimes by collating the reported number of side effects and treatment discontinuation rates. A total of 231 participants discontinued due to adverse events. The highest discontinuation rate due to adverse events was observed in the antimuscarinic group (12.7%). In comparison, all other treatment groups had a discontinuation rate due to adverse events of <4%.

Headache was the most frequently reported adverse effect with a1-blocker, PDE5-i and tolterodine-containing regimens, and nasopharyngitis was common with both PDE5-i and a1-blockers; both are consistent with the vasodilatory effects of these agents (52, 53). Dry mouth was the most frequent adverse effect with antimuscarinic combination therapy (54). Caution is warranted in men over 65 because of the potential effect of anticholinergic drugs on cognitive function (55, 56); the absence of reported cognitive impairment in the single antimuscarinic study (25) is likely to reflect its short duration (12 weeks), the relatively young participants (mean age 61.0 years) and the small sample size.

Serious cardiovascular events were rare across all treatment groups (see Results). This is consistent with systematic reviews showing that a1-blockers do not adversely affect the prognosis of heart failure (57) and that PDE5-i are not associated with an increased risk of cardiovascular events or mortality (58).

### Alpha-1 adrenergic receptor antagonists

Current EAU and AUA guidelines continue to recommend a1-blockers as the first-line therapy for men with moderate-to-severe LUTS, particularly where rapid symptom relief is desired (3, 4). The main mechanism of action is the reduction of prostate tone and bladder outflow obstruction by inhibiting the effect of noradrenaline on smooth muscle cells in the prostate (5). Pharmacological treatments in this review—including tamsulosin, alfuzosin, doxazosin, and silodosin—produced consistent improvements in IPSS (mean reduction 7.08 points) and Qmax (mean increase 2.49 mL/s). The mean reduction in IPSS observed with a1-blockers was numerically greater than that reported across the PDE5-i treatment arms. Whilst direct comparative conclusions cannot be drawn, these findings align with long-standing evidence suggesting rapid symptom relief through smooth muscle relaxation at the level of the prostate and bladder neck (5). The majority of the follow up was short term with the longest evaluation period lasting 52 weeks (41).

The observed improvement in Qmax is comparable with prior meta-analyses and supports the existing recommended guidelines for the use of alpha blockers in moderate to severe LUTS (5). The landmark Medical Therapy of Prostatic Symptoms (MTOPS) study, identified that the alpha blockers used alone or in combination with 5-alpha reductase inhibitors reduced clinical progression of BPO, incidence of acute urinary retention, and need for invasive therapy (59). This review is unable to draw meaningful conclusions regarding progression endpoints such as acute urinary retention or need for surgery as these were not reported in the evaluated studies. A reason for this could be the relatively short follow up period compared with the 4.5 year mean follow up in the MTOPS study (59). Thus, while symptomatic efficacy is confirmed, disease-modifying capacity remains unsupported.

### Phosphodiesterase type-5 inhibitors

PDE5-i, predominantly Tadalafil, demonstrated consistent improvement in IPSS (mean reduction 5.03 points), and an increase in Qmax (1.73 mL/s). These findings mirror pooled analyses supporting tadalafil 5 mg once daily as an effective therapy for LUTS, particularly in men with concomitant erectile dysfunction (6). However, many of the included PDE5-i trials specifically enrolled men with erectile dysfunction, and several were industry-sponsored registration trials of tadalafil 5 mg, which may limit the generalisability of these findings to men without erectile dysfunction.

PDE5-i inhibits the breakdown of cyclic guanosine monophosphate (cGMP), increasing availability of nitric oxide (NO) and therefore promoting smooth muscle relaxation. This results in vasodilation and relaxation of detrusor, prostate, and urethral muscles. Furthermore, these agents may alleviate LUTS through the modulation of spinal reflex pathways, enhancement of pelvic blood perfusion and oxygenation, and the reduction of chronic inflammation within the bladder and prostate (3). Participants noted improvements in their LUTS as reflected in the improved mean IPSS. Notably, one treatment arm using intermittent sildenafil reported slight symptom worsening, suggesting continuous daily therapy is likely required for sustained improvement in LUTS (38).

Across the reviewed studies, PDE5-i monotherapy generally demonstrated smaller mean improvements in PVR and IPSS than those reported for a1-blocker monotherapy and combination therapy. Improvements in Qmax and IPSS QoL were observed consistently across treatment classes. Differences between treatment approaches should be interpreted cautiously, as the heterogeneity of study populations, interventions, and follow-up durations precludes robust comparative assessment.

### Combination: a1-blocker + PDE5-i

Combination therapy with an a1-blocker plus a PDE5-i demonstrated the largest mean improvements in PVR, Qmax, IPSS, and IPSS-QoL across the reviewed studies. The mean increase in Qmax was 4.01 mL/s, which was greater than the average improvements reported in the monotherapy groups. These findings may reflect complementary mechanisms of action; however, direct comparisons between treatment classes are limited by substantial study heterogeneity.

Despite the encouraging findings, several limitations preclude the development of clinical recommendations. Many combination trials were relatively small, of limited duration, and insufficiently powered for superiority comparisons. Baseline prostate volume and LUTS phenotype were inconsistently reported, limiting assessment of which patient subgroups would derive the greatest benefit. Furthermore, long-term progression data were lacking. Combination therapy appears to be effective particularly in men with concomitant erectile dysfunction. However, its routine use cannot be universally endorsed without adequately powered randomised-controlled trials with extended follow up.

### Antimuscarinic combination therapy

Detrusor overactivity secondary to bladder outflow obstruction is a known consequence of BPO. Antimuscarinics inhibit the attachment of acetylcholine to M2 and M3 receptors in the detrusor muscle of the bladder. By reducing involuntary bladder spasms during the storage phase, antimuscarinics aim to increase bladder capacity and reduce the sensation of urgency (60).

Antimuscarinic therapy combined with a1-blocker demonstrated improvement in symptom scores and flow parameters but at the cost of higher discontinuation rates (12.7%) when compared with the other treatment groups. Acute urinary retention is an established adverse effect of antimuscarinic therapy (61). Although concerns regarding urinary retention were not substantiated in the small cohorts analysed, sample sizes were insufficient to exclude rare adverse events. The evidence base for antimuscarinic combination therapy was limited to a single study and a relatively small number of participants. Consequently, meaningful comparisons with other treatment classes cannot be made. These results should also be interpreted in the context of the patient population. In the TIMES trial, the benefit of adding tolterodine to tamsulosin was seen in men with overactive bladder symptoms (62). The improvement observed with antimuscarinic combination therapy in this review is therefore likely to reflect, at least in part, treatment of concomitant storage symptoms rather than of BPO itself. As baseline storage symptoms were not consistently reported, the subgroup most likely to benefit could not be defined. These findings support guideline recommendations permitting antimuscarinic use in carefully selected men with predominant storage symptoms and low baseline PVR, provided close monitoring is undertaken (3, 4).

### Future research

Emerging treatment options targeting diverse pathways including NO/cGMP signalling, TGF-*β*1 modulation, cannabinoid receptors, vitamin D analogues, botulinum toxin, and novel hormone-independent molecular targets demonstrate promising early efficacy but remain supported predominantly by preclinical or early-phase clinical data. Future adequately powered randomized trials with standardized patient-reported and functional outcomes are required to define their comparative effectiveness, safety, and potential role within the contemporary treatment algorithm for BPH/BPO.

Learning from this review, future studies should prioritise longer term follow up (minimum ≥24 months), quantitative clinical endpoints (urinary retention, surgery), stratification by prostate volume and symptom phenotype, standardised dosing and reporting frameworks, and adequately powered superiority trials comparing monotherapy vs. combination therapy.

With the ageing population and increasing healthcare burden of BPO on clinical services, further research is required to identify the long-term impact of non-hormonal pharmacological therapies. Studies with a longer term follow up of ≥24 months are needed to assess if the benefits to patients symptomatically (IPSS) and clinically (Qmax and PVR) and sustained with regular long-term use. Furthermore, quantitative clinical end points, such as acute retention and need for surgical interventions, would help clinicians in the decision-making process in the management of BPO**.**

### Methodological considerations

Several methodological limitations should be acknowledged. First, heterogeneity in dosing regimens, treatment duration, and baseline symptom severity limited direct cross-study comparisons. Second, the majority of trials relied on short-term surrogate endpoints (IPSS, Qmax, PVR), with minimal reporting of progression outcomes. Third, detailed reporting of allocation concealment, blinding, and intention-to-treat analysis was inconsistent, raising the possibility of bias. Given the subjective nature of symptom-based endpoints, inadequate blinding may inflate treatment effects. Furthermore, the within-arm changes summarised in this review include the placebo response, which is substantial in LUTS trials, and do not represent placebo-adjusted treatment effects. Differences of a few IPSS points between groups should also be considered against the minimal clinically important difference of approximately 3 points (63).

Results were summarised by drug class, combining different agents, doses and formulations within each class. Although this provides an overview of the contemporary evidence, agents within a class are not interchangeable, and the class-level summaries should not be interpreted as pooled estimates of efficacy. In addition, the absolute changes shown in Figures 3–6 were not adjusted for baseline severity. Because men with more severe baseline symptoms or lower baseline flow rates tend to show larger absolute improvements, apparent differences between treatment groups may partly reflect differences in baseline characteristics rather than differences in drug effect.

**Figure 3:**
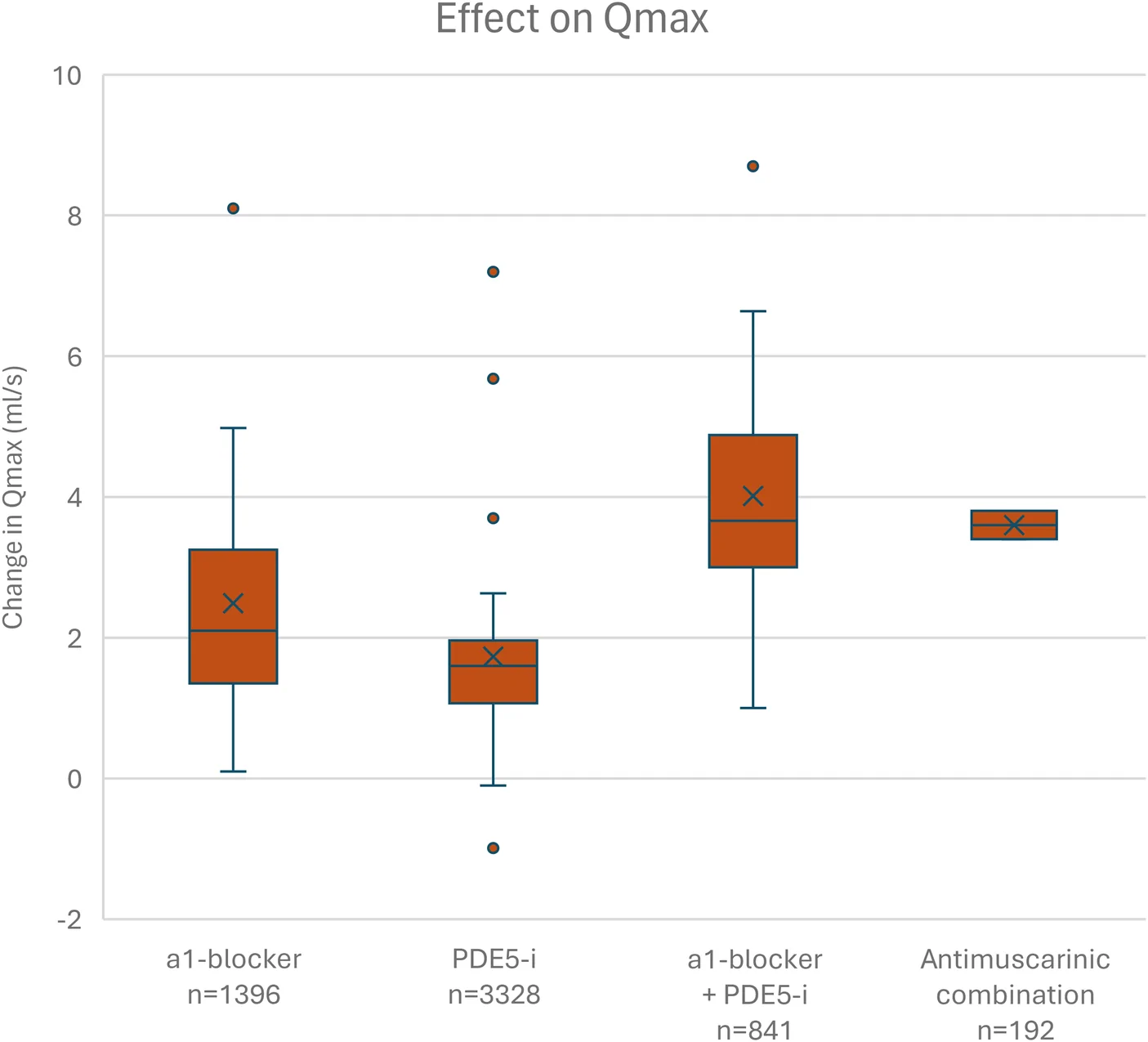
Box and whisker plot graphs that illustrate the reported mean changes of post-void residual volume (PVR) for each treatment group. Interquartile range (IQR) of these means is represented by box size, the line within the box represents the median result and the whiskers show the minimum and maximum result within 1.5× IQR from quartile 1 and quartile 3. Outlier results are represented as dots. Mean result denoted by X. PDE5-i = phosphodiesterase type 5 inhibitor, a1-blocker = 1-adrenergic receptor antagonist. *n* = number of participants who received this intervention for which change in PVR was reported. Values are unadjusted arm-level mean changes from baseline and are not adjusted for differences in baseline severity, agent, dose or follow-up duration; the plots are descriptive and are not intended as a comparison of efficacy between treatment groups.

**Figure 4:**
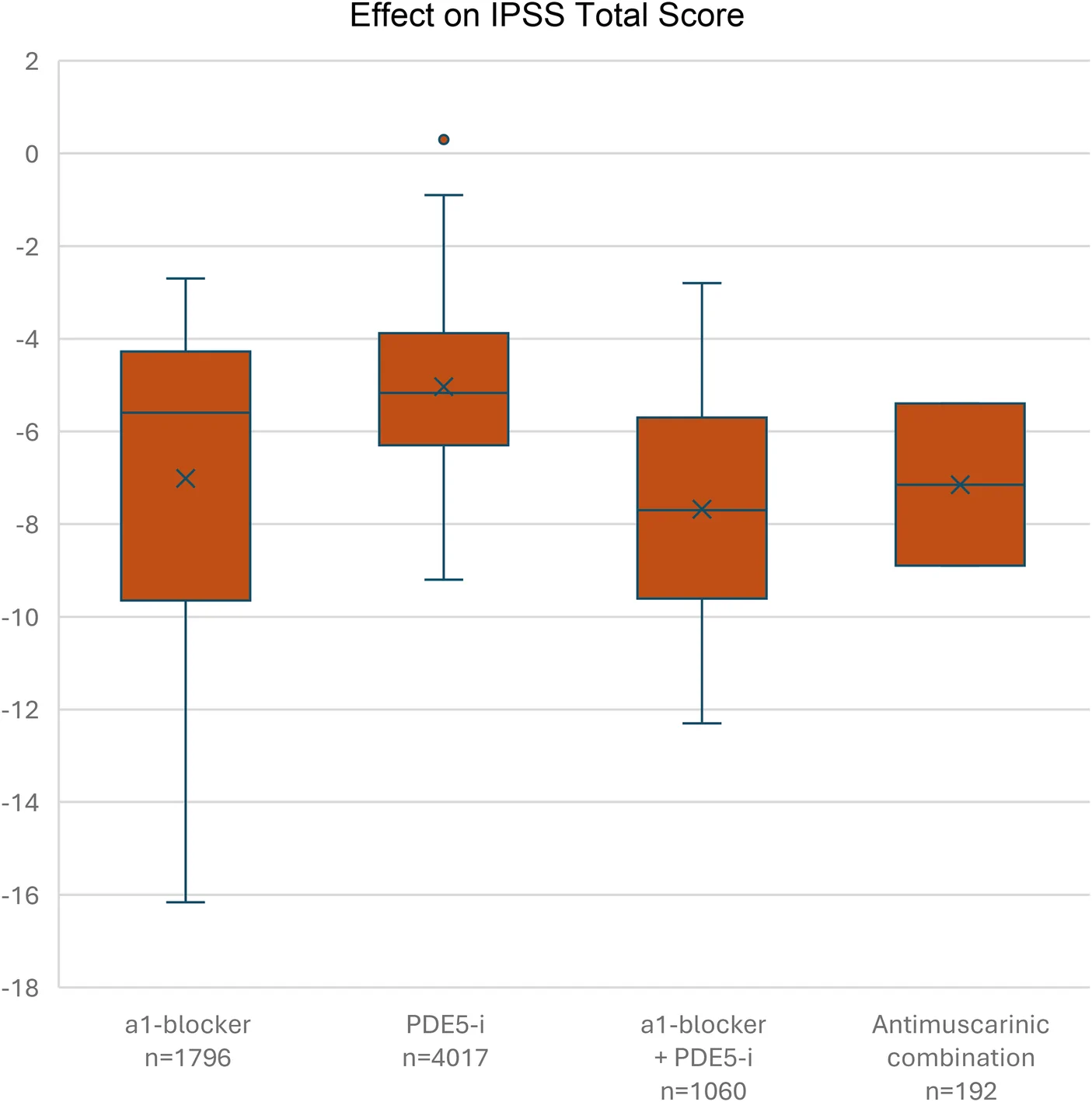
Box and whisker plot graphs that illustrate the reported mean change in maximum urinary flow rate (Qmax) from each of the treatment groups. Interquartile range (IQR) of these means is represented by box size, the line within the box represents the median result and the whiskers show the minimum and maximum result within 1.5× IQR from quartile 1 and quartile 3. Outlier results are represented as dots. Mean result denoted by X. PDE5-i = phosphodiesterase type 5 inhibitor, a1-blocker = 1-adrenergic receptor antagonist. *n* = number of participants who received this intervention for which change in Qmax was reported. Values are unadjusted arm-level mean changes from baseline and are not adjusted for differences in baseline severity, agent, dose or follow-up duration; the plots are descriptive and are not intended as a comparison of efficacy between treatment groups.

**Figure 5:**
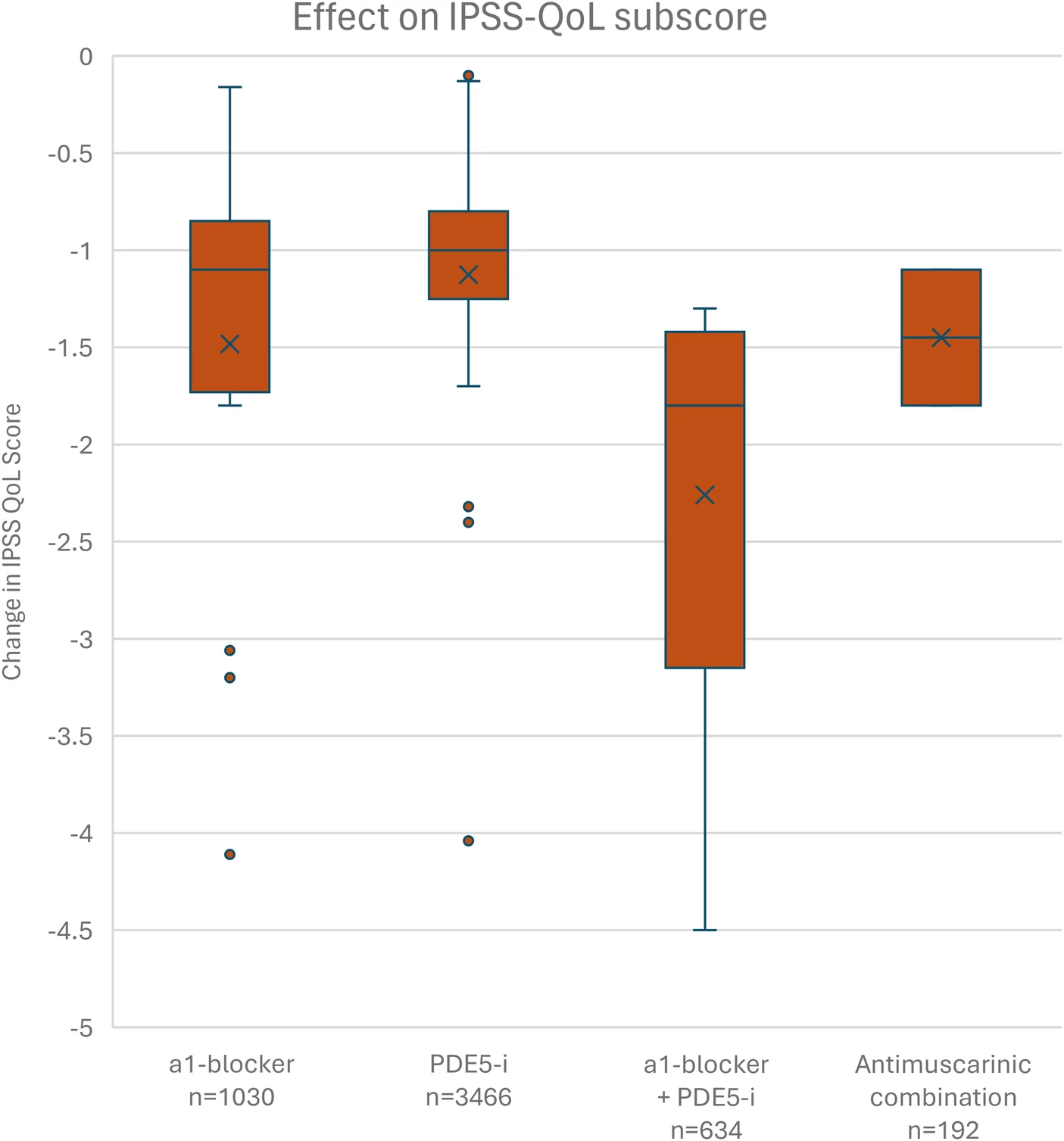
Box and whisker plot graphs that illustrate the reported mean change in International Prostate Symptom Score (IPSS) total for each of the treatment groups. Interquartile range (IQR) of these means is represented by box size, the line within the box represents the median result and the whiskers show the minimum and maximum result within 1.5× IQR from quartile 1 and quartile 3. Outlier results are represented as dots. Mean result denoted by X. PDE5-i = phosphodiesterase type 5 inhibitor, a1-blocker = 1-adrenergic receptor antagonist. *n* = number of participants who received this intervention for which change in IPSS total was reported. Values are unadjusted arm-level mean changes from baseline and are not adjusted for differences in baseline severity, agent, dose or follow-up duration; the plots are descriptive and are not intended as a comparison of efficacy between treatment groups.

**Figure 6:**
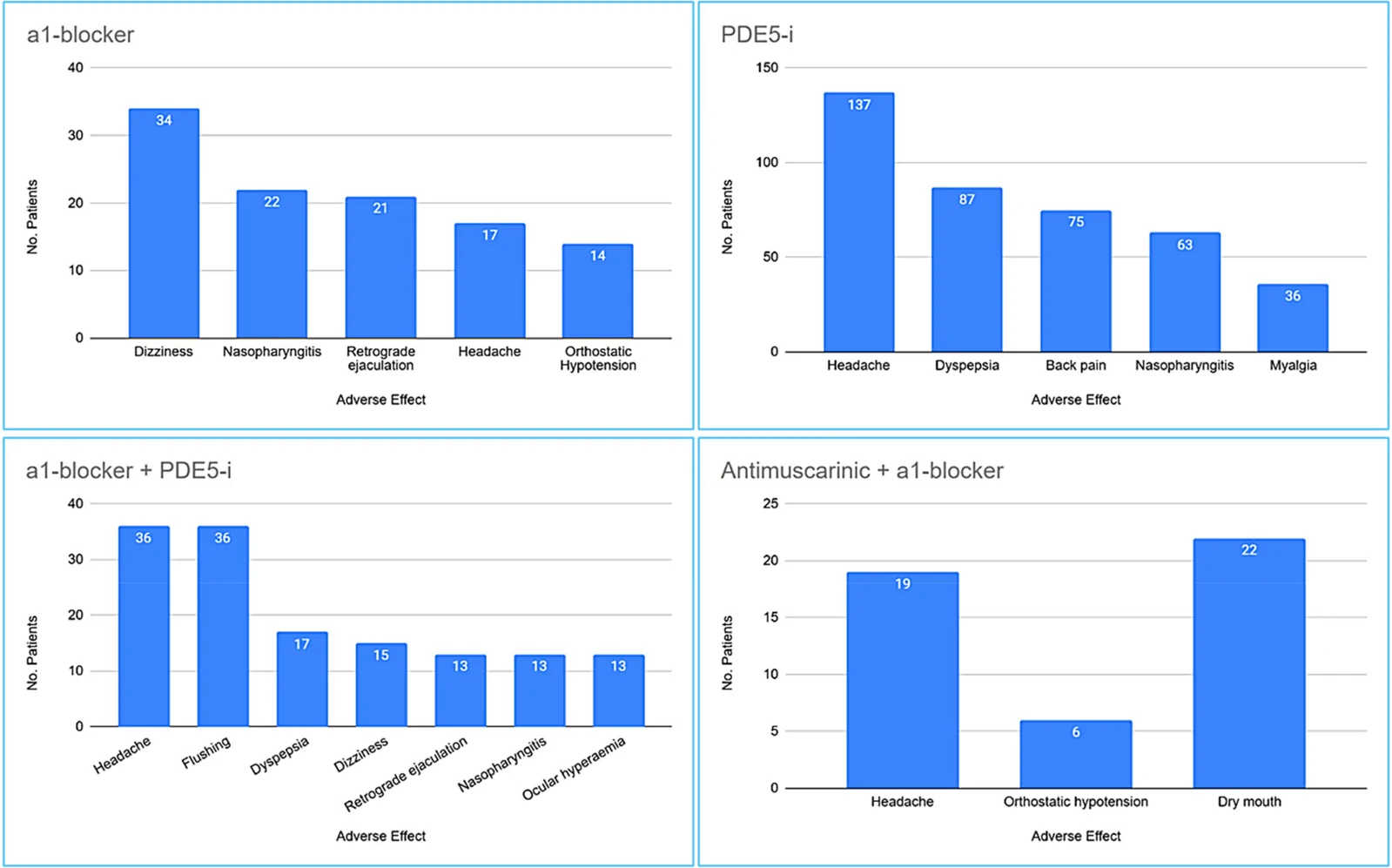
Box and whisker plot graphs that illustrate the reported mean change in International Prostate Symptom Score—Quality of Life subscore (IPSS-QoL) for each of the treatment groups. Interquartile range (IQR) of these means is represented by box size, the line within the box represents the median result and the whiskers show the minimum and maximum result within 1.5× IQR from quartile 1 and quartile 3. Outlier results are represented as dots. Mean result denoted by X. PDE5-i = phosphodiesterase type 5 inhibitor, a1-blocker = 1-adrenergic receptor antagonist. *n* = number of participants who received this intervention for which change in IPSS-QoL was reported. Values are unadjusted arm-level mean changes from baseline and are not adjusted for differences in baseline severity, agent, dose or follow-up duration; the plots are descriptive and are not intended as a comparison of efficacy between treatment groups.

A further limitation relates to the search strategy. Restricting the search to studies published from 2005 onwards, together with the use of focused key words, means that the pivotal a1-blocker registration trials, most of which were published before 2005, were not included, and some later trials may also have been missed. The a1-blocker results presented here therefore reflect contemporary trials, in which a1-blockers were most often used as active comparators or as part of combination therapy, rather than a comprehensive synthesis of a1-blocker efficacy, which is well established in earlier meta-analyses and guidelines (3–5). Not all non-hormonal pharmacological treatment options are covered in this review, and the review was registered retrospectively.

The predominance of positive findings across small trials also raises the possibility of publication bias. Additionally, trial populations were relatively young (mean age 62.6 years). These participants are likely healthier than real-world cohorts which potentially limits external validity.

## Conclusion

This systematic review of 42 contemporary studies involving 10,066 participants shows that a1-blockers, PDE5-i, and combination therapies were associated with symptomatic improvement in men with LUTS secondary to BPO (10–51). While each treatment class demonstrated improvements in LUTS-related outcomes, combination therapy with a1-blockers and PDE5-i showed the largest mean improvements across the reviewed studies in IPSS, IPSS Qol, PVR, and Qmax. Specifically, the combination of a1-blocker and PDE5-i was associated with a mean Qmax increase of 4.01 mL/s, compared to the mean increases of 2.49 mL/s and 1.73 mL/s for a1-blocker and PDE5-i monotherapy, respectively. However, owing to the heterogeneity in study design, patient populations, treatment regimens, and follow-up duration, these findings should be interpreted as descriptive observations rather than evidence of comparative superiority.

While current treatment regimes demonstrate a high degree of tolerability and low discontinuation rates, the existing evidence base is constrained by short-term follow up and reliance on subjective outcome measures. Long-term disease modifying capacity and impact on quantitative outcomes such as acute urinary retention or the need for secondary surgical intervention remains largely unsupported. To redefine the standard of care, future research must shift towards rigorously designed, adequately powered trials with extended follow up (≥24 months) and a greater emphasis on patient characteristics to better personalise treatment for the ageing global population.
