# Supplementary material for: Non-hormonal pharmacological treatments for benign prostatic obstruction: a systematic review of trials published between 2005 and 2025
Source: Front Surg. 2026 Oct 28;13:1898220. doi: 10.3389/fsurg.2026.1898220 (PMC13752569; doi:10.3389/fsurg.2026.1898220)
Supplement: Supplementary file 1 [file Table1.docx]

**Supplementary Table 1**

| **Authors** | **Year published** | **Type of study** | **Centre (s)** | **Drug and daily dosage *** | **Mean age** | **No. randomised**  **participants** | **No. of participants completed study** | **Overall number of participants included in study** | **Co-morbidities** |
| --- | --- | --- | --- | --- | --- | --- | --- | --- | --- |
| Zahir et al. | 2023 | Open-label, single-arm clinical trial | Multicentre (n=3) | Sildenafil 50mg | 59.8 | 40 | 33 | 33 | NR |
|  |  |  |  | Tadalafil 5mg | 59.8 | 40 | 33 |  |  |
| Gupta et al. | 2023 | Retrospective observational study | Multicentre | Alfuzosin 10mg | 59.74 | 166 | 156 | 138 | DM, HTN, cardiovascular disease, renal disease, hyperlipidaemia, obesity |
|  |  |  |  | Silodosin 8mg | 59.95 | 67 | 65 |  |  |
|  |  |  |  | Tamsulosin 0.4mg | 68.71 | 67 | 67 |  |  |
|  |  |  |  | Prazosin 0.5mg | 62.46 | 32 | 32 |  |  |
| Sevach et al. | 2023 | Prospective comparative study | Single centre | Tamsulosin 0.4mg | 64.57 | 50 | 50 | 100 | NR |
|  |  |  |  | Silodosin 8mg | 63.15 | 50 | 50 |  |  |
| AbdelRazek et al. | 2022 | Randomised, open label, three-arm trial (no control) | Single centre | Tadalafil 5mg | 61.7 | 124 | 101 | 308 | NR |
|  |  |  |  | Silodosin 8mg | 62.9 | 126 | 102 |  |  |
|  |  |  |  | Tadalafil 5mg + Silodosin 8mg | 62.6 | 130 | 105 |  |  |
| Singh et al. | 2020 | Parallel-group randomised clinical trial | Single centre | Tadalafil 5mg | 62.56 | 50 | 50 | 100 | NR |
|  |  |  |  | Tamsulosin 0.4mg | 63.44 | 50 | 50 |  |  |
| Atan et al. | 2019 | Retrospective observational study | Single centre | Tadalafil  5mg | 61 | 23 | 23 | 23 | NR |
| Negoro et al. | 2019 | Randomised, double-blind, placebo-controlled crossover trial | Multicentre (n=2) | Tadalafil  5 mg | 73 | 13 | 13 | 26 | NR |
|  |  |  |  | Placebo | 70 | 13 | 13 |  |  |
| Xue et al. | 2018 | Randomised controlled trial | Single centre | Tamsulosin 8mg | 69.5 | 85 | 80 | 148 | HTN, CAD, DM |
|  |  |  |  | Placebo | 68.5 | 85 | 68 |  |  |
| Zhang et al. | 2018 | Randomised, double-blind, placebo-controlled trial | Multicentre (n=40) | Tadalafil 5mg | 61.8 | 363 | 346 | 857 | NR |
|  |  |  |  | Tamsulosin 0.2mg | 61.9 | 185 | 174 |  |  |
|  |  |  |  | Placebo | 61.8 | 361 | 337 |  |  |
| Urakami et al. | 2018 | Randomised, open label, non-inferiority comparative trial | Single centre | Tadalafil 5mg | 70 | 38 | 31 | 56 | NR |
|  |  |  |  | Solifenacin 5mg | 72 | 37 | 25 |  |  |
| Matsukawa et al. | 2017 | Open-label prospective cohort study | Single centre | Tadalafil 5mg | 70.2 | 80 | 71 | 71 | DM (n=12), HTN (n=39), hyperlipidaemia (n=26) |
| Kim et al. | 2017 | Randomised, double-blind, active-controlled, parallel-group trial | Multicentre (n=15) | Tamsulosin 0.4mg + Tadalafil 5 mg | 61.84 | 169 | 153 | 479 | Smoker (n=25), HTN (n=57), DM (n=23), hyperlipidaemia (n=35) |
|  |  |  |  | Tamsulosin 0.2mg + Tadalafil 5 mg | 62.58 | 170 | 164 |  | Smoker (n=34), HTN (n=73), DM (n=35), hyperlipidaemia (n=39) |
|  |  |  |  | Tadalafil 5 mg | 61.93 | 171 | 162 |  | Smoker (n=18), HTN (n=57), DM (n=22), hyperlipidaemia (n=39) |
| Fawzi et al. | 2017 | Randomised, double-blind, placebo-controlled trial | Single centre | Tamsulosin 0.4mg + Sildenafil 25mg | 65.8 | 75 | 63 | 131 | NR |
|  |  |  |  | Tamsulosin 0.4mg + Placebo | 66.3 | 75 | 68 |  |  |
| Yoshida et al. | 2017 | Randomised, open label, parallel-group trial | Multicentre (n=8) | Silodosin 8mg | 70.5 | 95 | 93 | 188 | HTN, hyperlipidaemia, DM, OAB |
|  |  |  |  | Tadalafil 5mg | 69.7 | 97 | 95 |  |  |
| Ko et al. | 2017 | Randomised, double-blind, placebo-controlled trial | Multicentre (n=3) | Sildenafil 50mg | 60.5 | 43 | 37 | 147 | NR |
|  |  |  |  | Sildenafil 100mg | 59.8 | 43 | 3 |  |  |
|  |  |  |  | Sildenafil 200mg | 61.4 | 44 | 34 |  |  |
|  |  |  |  | Placebo | 61.3 | 44 | 37 |  |  |
| Cao et al. | 2016 | Randomised, open-label, comparative study | Single centre | Doxazosin 4mg + Tolterodine 4mg | 61.4 | 110 | 97 | 192 | NR |
|  |  |  |  | Tamsulosin 0.2mg + Tolterodine 4mg | 60.5 | 110 | 95 |  |  |
| Karami et al. | 2016 | Randomised, single-blind, controlled trial | Single centre | Tadalafil 20mg | 68.2 | 61 | 60 | 177 | NR |
|  |  |  |  | Tamsulosin 0.4mg | 68.5 | 61 | 59 |  |  |
|  |  |  |  | Tadalafil 20mg + Tamsulosin 0.4mg | 67.9 | 61 | 58 |  |  |
| Singh et al. | 2014 | Randomised, open-label, three-arm trial | Single centre | Tamsulosin 0.4 mg | 59.5 | 45 | 43 | 125 | NR |
|  |  |  |  | Tadalafil 10mg | 63.42 | 44 | 40 |  |  |
|  |  |  |  | Tamsulosin 0.4mg + Tadalafil 10mg | 61.92 | 44 | 42 |  |  |
| Takeda et al. | 2014 | Randomised, double-blind, placebo-controlled, parallel-group trial | Multicentre (n=39) | Tadalafil 5mg | 60.8 | 306 | 292 | 585 | NR |
|  |  |  |  | Placebo | 60.9 | 304 | 293 |  |  |
| Abdel-Kader et al. | 2013 | Randomised, open-label trial | Single centre | Sildenafil 50mg | NR | 50 | 50 | 150 | NR |
|  |  |  |  | Doxazosin 2mg | NR | 50 | 50 |  |  |
|  |  |  |  | Sildenafil 50mg + Doxazosin 2mg | NR | 50 | 50 |  |  |
| Kumar et al. | 2013 | Randomised, open-label controlled trial | Single centre | Alfuzosin 10mg | 60.1 | 25 | 25 | 75 | NR |
|  |  |  |  | Tadalafil 10mg | 63.1 | 25 | 25 |  |  |
|  |  |  |  | Alfuzosin 10mg + Tadalafil 10mg | 62.6 | 25 | 25 |  |  |
| Egerdie et al. | 2012 | Randomised, double-blind, placebo-controlled trial | Multicentre (n=54) | Tadalafil 2.5mg | 62.2 | 198 | 172 | 526 | NR |
|  |  |  |  | Tadalafil 5mg | 62.5 | 208 | 184 |  |  |
|  |  |  |  | Placebo | 62.9 | 200 | 170 |  |  |
| Oelke et al. | 2012 | Randomised, double-blind, placebo- and active-controlled trial | Multicentre (n=44) | Tadalafil 5mg | 63.5 | 171 | 156 | 454 | NR |
|  |  |  |  | Tamsulosin 0.4mg | 63.5 | 168 | 150 |  |  |
|  |  |  |  | Placebo | 63.7 | 172 | 148 |  |  |
| Yokoyama et al. | 2012 | Randomised, double-blind, placebo-controlled, parallel-group trial | Multicentre (n=34) | Tadalafil 2.5mg | 63.7 | 151 | 136 | 561 | NR |
|  |  |  |  | Tadalafil 5mg | 62.3 | 155 | 137 |  |  |
|  |  |  |  | Tamsulosin 0.2mg | 62.6 | 152 | 143 |  |  |
|  |  |  |  | Placebo | 63.7 | 154 | 145 |  |  |
| Madani et al. | 2012 | Randomised, double-blind, placebo-controlled trial | Single centre | Tadalafil 10mg | 64.4 | 66 | 60 | 122 | NR |
|  |  |  |  | Placebo | 64.87 | 66 | 62 |  |  |
| Regadas et al. | 2012 | Randomised, double-blind, placebo-controlled trial | Single centre | Tamsulosin 0.4mg + Tadalafil 5mg | 61.6 | 20 | 20 | 40 | NR |
|  |  |  |  | Tamsulosin 0.4mg + placebo | 59.2 | 20 | 20 |  |  |
| Shirakawa et al. | 2012 | Randomised, open-label, controlled trial | Multicentre | Silodosin 8mg | 70 | 35 | 34 | 112 | NR |
|  |  |  |  | Naftopidil 50mg | 70.5 | 33 | 32 |  |  |
|  |  |  |  | Switched from Tamsulosin to Silodosin 8mg | 72.5 | 26 | 22 |  |  |
|  |  |  |  | Switched from Tamsulosin to Naftopidil 50mg | 70.5 | 27 | 24 |  |  |
| Gacci et al. | 2012 | Randomised, double-blind, placebo-controlled trial | Single centre | Tamsulosin 0.4mg + Vardenafil 10mg | 66.3 | 30 | 30 | 59 | NR |
|  |  |  |  | Tamsulosin 0.4mg + Placebo | 69.6 | 30 | 29 |  |  |
| Jin et al. | 2011 | Randomised, open-label, parallel-group, non-inferiority trial | Multicentre (n=5) | Doxazosin GITS 4mg + Sildenafil 25-100mg PRN | 61.7 | 168 | 137 | 203 | NR |
|  |  |  |  | Sildenafil 25-100 mg PRN | 60.8 | 82 | 66 |  |  |
| Öztürk et al. | 2011 | Randomised, open-label, parallel-group trial | Single centre | Alfuzosin XL 10mg | 60.2 | 50 | 47 | 92 | HTN (n=18), DM (n=13), CAD (n=6) |
|  |  |  |  | Alfuzosin XL 10mg + Sildenafil 50mg | 60.2 | 50 | 45 |  |  |
| Porst et al. | 2011 | Randomised, double-blind, placebo-controlled, parallel-group trial | Multicentre (n=28) | Tadalafil 5mg | 65.1 | 161 | 148 | 300 | NR |
|  |  |  |  | Placebo | 64.6 | 164 | 152 |  |  |
| Donatucci et al. | 2011 | Open-label extension study of a prior randomised, double-blind, placebo-controlled trial | Multicentre (n=44) | Tadalafil 5mg | 62.66 | 427 | 299 | 299 | NR |
| Kim et al. | 2011 | Randomised, double-blind, placebo- and active-controlled trial | Multicentre (n=10) | Tadalafil 5mg | 61.2 | 51 | 48 | 143 | NR |
|  |  |  |  | Tamsulosin 200mcg | 61.5 | 49 | 48 |  |  |
|  |  |  |  | Placebo | 62.2 | 51 | 47 |  |  |
| Dmochowski et al. | 2010 | Randomised, double-blind, placebo-controlled trial | Multicentre (n=20) | Tadalafil 20mg | 58.2 | 99 | 83 | 172 | NR |
|  |  |  |  | Placebo | 59 | 101 | 89 |  |  |
| Tuncel et al. | 2009 | Randomised, three-arm trial | Single centre | Sildenafil citrate 25mg four times a week | 58.8 | 20 | 20 | 60 | NR |
|  |  |  |  | Tamsulosin 0.4mg once a day |  | 20 | 20 |  |  |
|  |  |  |  | Sildenafil citrate 25mg four times a week + Tamsulosin 0.4mg once a day |  | 20 | 20 |  |  |
| Liguori et al. | 2009 | Randomised, open-label, three-arm trial | Multicentre (n=5) | Alfuzosin 10mg | 61.3 | 22 | 18 | 58 | DM, HTN, IHD |
|  |  |  |  | Tadalafil 20mg | 60.8 | 21 | 19 |  |  |
|  |  |  |  | Alfuzosin 10mg + Tadalafil 20mg | 61.5 | 23 | 21 |  |  |
| Chung et al. | 2009 | Prospective, open-label, non-comparative study | Multicentre (n=2) | Udenafil 100mg once a week (co-administered with alpha1-blocker: doxazosin, tamsulosin, or alfuzosin) | 58.4 | 120 | 82 | 82 | HTN (n=41) |
| Bechara et al. | 2008 | Randomised, double-blind, crossover trial | Single centre | Tamsulosin 0.4mg + Tadalafil 20mg | 63.7 | 30 | 27 | 27 | NR |
|  |  |  |  | Tamsulosin 0.4mg + placebo | 63.7 | 30 | 27 |  |  |
| Roehrborn et al. | 2008 | Randomised, double-blind, placebo-controlled trial | Multicentre (n=92) | Placebo | 61.75 | 212 | 185 | 886 | NR |
|  |  |  |  | Tadalafil 2.5mg | 62.03 | 209 | 182 |  |  |
|  |  |  |  | Tadalafil 5mg | 61.95 | 212 | 182 |  |  |
|  |  |  |  | Tadalafil 10mg | 62.22 | 216 | 175 |  |  |
|  |  |  |  | Tadalafil 20mg | 62.55 | 209 | 162 |  |  |
| Stief et al. | 2008 | Randomised, double-blind, placebo-controlled trial | Multicentre (n=16) | Vardenafil 20 mg | 56.5 | 109 | 105 | 215 | ED |
|  |  |  |  | Placebo | 55.4 | 113 | 110 |  |  |
| Kaplan et al. | 2007 | Randomised, open-label, three-arm trial | Single centre | Alfuzosin 10mg | 62.6 | 20 | 18 | 55 | DM, HTN, IHD |
|  |  |  |  | Sildenafil 25mg | 64.5 | 21 | 19 |  |  |
|  |  |  |  | Alfuzosin 10mg + Sildenafil 25mg | 63.1 | 21 | 18 |  |  |
| McVary et al. | 2007 | Randomised, double-blind, placebo-controlled, parallel-group trial | Multicentre (n=21) | Tadalafil 5mg | 62 | 138 | 129 | 262 | NR |
|  |  |  |  | Placebo | 61 | 143 | 133 |  |  |

**Table 8:** Study characteristics of included articles (CAD = coronary artery disease, DM = diabetes mellitus, ED = erectile dysfunction, HTN = hypertension, IHD = ischaemic heart disease, mg = milligram, NR = not recorded, OAB = overactive bladder)

* Doses are per day, unless specified directly on the table
