# Supplementary material for: Non-hormonal pharmacological treatments for benign prostatic obstruction: a systematic review of trials published between 2005 and 2025
Source: Front Surg. 2026 Oct 28;13:1898220. doi: 10.3389/fsurg.2026.1898220 (PMC13752569; doi:10.3389/fsurg.2026.1898220)
Supplement: Supplementary file 2 [file Table2.docx]

**Supplementary Table 2: risk of bias analysis for included studies.**
